# Editorial: Ketogenic diets for cognitive and behavioral function

**DOI:** 10.3389/fnut.2024.1463796

**Published:** 2024-08-07

**Authors:** Jose Enrique de la Rubia Ortí, María Pilar García-Pardo, María Cuerda-Ballester, Gloria Castellano-Estornell, María Benlloch

**Affiliations:** ^1^Department of Basic Biomedical Sciences, Catholic University of Valencia, Valencia, Spain; ^2^Department of Psychology and Sociology, University of Zaragoza, Teruel, Spain; ^3^Department of Nursing, Catholic University of Valencia San Vicente Martir, Valencia, Spain; ^4^Translational Research Center San Alberto Magno (CITSAM), Catholic University of Valencia, Valencia, Spain

**Keywords:** ketogenic diet, incurable diseases, inflammation, neurodegenerative diseases, ketone metabolism

Neurodegenerative diseases represent a broad set of pathologies, based on the progressive death of neurons in different regions of the nervous system. Given the complexity of the nervous system, manifestations depending on the affected area are numerous and diverse. Prominent among these is the potential impairment of movement, memory, language, cognition, and learning, causing patients to suffer deterioration in their quality of life and autonomy.

However, despite being incurable diseases, in recent years the importance of diet in improving the prognosis of these pathologies has been highlighted. Among these, ketogenic diets stand out as they are able to counteract the pathogenic processes that characterize neurodegeneration, such as high oxidative stress, inflammation, and energy abnormalities at the mitochondrial level. Therefore, they are increasingly being used as complements to treatments, especially because of the benefits evidenced at the cognitive and emotional levels.

In this sense, this Research Topic aims to present some of the latest scientific works that provide information on different facets of ketogenic diets. Specifically, four research articles were included, of which three are reviews and a mini review (Kong et al.; Cecchi et al.; de la Rubia Ortí et al.), and one is original research (Wiers et al.).

In the first paper included in this Research Topic, Kong et al., through a review analyze the impact of ketogenic diets on substance use disorders (SUD), by means of a review. Reducing carbohydrate intake through ketogenic diet can have a positive effect. To delve into this potential benefit, it is assumed that ketone bodies can compensate for glucose metabolism disorders caused by alcohol consumption by increasing the ketone metabolism. Thus, the aim was to reduce withdrawal symptoms. It is also worth noting that SUD is specifically linked to mitochondrial damage, oxidative stress, inflammation, glial dysfunction, and intestinal microbial disorders, which are pathogenic mechanisms that can be reversed by ketogenic diets. This is why this study delves into these benefits, considering the side effects that have been associated with consuming these diets, such as metabolic abnormalities, increased risk of malnutrition, and gastrointestinal symptoms.

Similarly, Wiers et al. addressed in a highly interesting way how a ketogenic diet can modify the desire to drink alcohol in people with alcohol use disorder (AUD) undergoing abstinence for over 3 weeks. To do this, it is based on aspects already considered in the review by Kong et al., and specifically on the fact that acute alcohol consumption changes brain energy from glucose to acetate (an alcohol metabolite), and this change is maintained over time after abandoning the habit. In this sense, the authors propose that promoting ketone body production through the diet, which is structurally very similar to acetate, can suppress the signs and symptoms of alcohol abstinence, craving for alcohol, and the possibility of relapse that occurs precisely as a result of low acetate availability. After the trial, it was observed that when comparing the effect during that time period of consuming a ketogenic diet vs. a standard American diet, the desire to drink alcohol decreased over the 3 weeks of abstinence only in the group of patients who followed the ketogenic diet.

Another study of interest on this Research Topic was by Cecchi et al. In the review by these authors, the benefits of farmaconutrition are brilliantly analyzed, combining personalized pharmacological treatment with a designed diet to synergize the effects of drugs in the treatment of pediatric gliomas. It should be noted that pediatric gliomas represent the most common brain tumor in children, which gives greater significance to the results and evidence provided by the authors in relation to the benefits of these diets.

Finally, the Research Topic provides a mini review carried out by de la Rubia Ortí et al., on the possible role of ketogenic diets in multiple sclerosis (MS), which represents the most disabling neurodegenerative disease in young people. This work aims to update the topic and discuss the potential impact of ketogenic diets on anxiety and depression through the modulation of glutamate activity. After analyzing the main findings, ketogenic diets seem to represent an alternative source of blood ketone bodies for improving glutamate activity. According to the authors, this benefit is mediated by reducing obesity, which is associated with insulin resistance and dyslipidemia, and finally with central inflammation. This would improve synaptic glutamate activity and decrease extrasynaptic activity, which in excess has been related to functional disability and the presence of anxiety and depression.

## Author contributions

JR: Conceptualization, Data curation, Formal analysis, Funding acquisition, Investigation, Methodology, Project administration, Resources, Software, Supervision, Validation, Visualization, Writing – original draft, Writing – review & editing. MG-P: Conceptualization, Data curation, Formal analysis, Funding acquisition, Investigation, Methodology, Project administration, Resources, Software, Supervision, Validation, Visualization, Writing – original draft, Writing – review & editing. MC-B: Conceptualization, Data curation, Formal analysis, Funding acquisition, Investigation, Methodology, Project administration, Resources, Software, Supervision, Validation, Visualization, Writing – original draft, Writing – review & editing. GC-E: Conceptualization, Data curation, Formal analysis, Funding acquisition, Investigation, Methodology, Project administration, Resources, Software, Supervision, Validation, Visualization, Writing – original draft, Writing – review & editing. MB: Conceptualization, Data curation, Formal analysis, Funding acquisition, Investigation, Methodology, Project administration, Resources, Software, Supervision, Validation, Visualization, Writing – original draft, Writing – review & editing.

